# Combinatorial Effects of Boron Compounds on Immunophenotypic Modulation of Mouse Hematopoietic Stem Cell Subsets Ex Vivo

**DOI:** 10.3390/metabo16060382

**Published:** 2026-05-31

**Authors:** Fatih Kocabaş, Eray Esendir, Neslihan Meriç

**Affiliations:** 1 Department of Molecular Biology and Genetics, Faculty of Engineering and Natural Sciences, Istanbul Atlas University, 34403 İstanbul, Türkiye; 2 Institute of Pharmacology and the Gaston H. Glock Research Laboratories for Exploratory Drug Development, Center of Physiology and Pharmacology, Medical University of Vienna, 1090 Wien, Austria; eray.esendir@meduniwien.ac.at; 3 Department of Molecular Biology and Genetics, Faculty of Engineering and Natural Sciences, Kütahya Health Sciences University, 43100 Kütahya, Türkiye; neslihan.meric@ksbu.edu.tr

**Keywords:** boric acid (BA), sodium pentaborate pentahydrate (NaB), hematopoietic stem cells (HSCs), lineage-negative (Lin−) cells, sodium 2-pentaborate pentahydrate-8 (Na2B8)

## Abstract

Background/Objectives: Hematopoietic stem cells (HSCs) sustain lifelong blood cell production and hold therapeutic promise, yet their ex vivo expansion remains constrained by an incomplete understanding of the metabolic and cellular mechanisms governing self-renewal. In this study, we investigated whether boron compounds boric acid (BA), sodium pentaborate pentahydrate (NaB), and sodium 2-pentaborate pentahydrate-8 (Na2B8) can promote the expansion of mouse HSCs by modulating key stem cell populations linked to metabolic fitness. Methods: Lineage-negative (Lin−) cells were magnetically isolated and treated with each boron compound for four days, followed by flow cytometric analysis of c-Kit, Sca-1, Lin-c-Kit+Sca-1+ (LSK), and LSKCD34^Low^ HSC-enriched subsets. Results: Our results show that boron derivatives exert distinct effects on these cellular markers. Notably, NaB treatment significantly increased the Lin-Sca-1+ cell fraction, while Na2B8 elevated both LSK and LSKCD34^Low^ ratios. Furthermore, the BA+NaB combination produced a statistically significant proliferative effect on Sca-1+ and c-Kit+ (CD117) cells. Conclusions: These findings indicate that specific boron compounds enhance ex vivo HSC expansion through yet-to-be-defined mechanisms that underpin HSC self-renewal. Further mechanistic studies are warranted to delineate the precise metabolic targets, but these results highlight boron compounds as promising tools for improving HSC expansion strategies.

## 1. Introduction

The proliferation and differentiation of HSCs are significantly influenced by the Lin-cell population in mice, especially Lin-c-Kit+Sca-1+ cells. Hematopoietic stem and progenitor cells (HSPCs) include Lin-cells, which are enriched for HSCs but lack lineage-specific markers. In the fields of regenerative medicine and HSC biology, the Lin-c-Kit+Sca-1+ phenotype has been thoroughly investigated. These cells are crucial for preserving hematopoiesis and fostering tissue regeneration because of their demonstrated high capacity for self-renewal and multilineage differentiation [1,2,3]. The importance of Lin-cells in the in vitro proliferation of mouse HSCs has been emphasized in a number of studies. For instance, it has been demonstrated that prolonged ex vivo proliferation of LSK cells results from the forced expression of Sall4 isoforms [3]. The frequency of Long Term (LT)-HSCs and Short Term (ST)-HSCs increased when PTPN13 or β-catenin was silenced, but their proliferative activity decreased and their quiescence increased [4]. These emphasize the significant influence of specific genes and proteins in controlling the proliferation of Lin− cells within the hematopoietic stem cell (HSC) population. Research has also underscored the hierarchical structure of hematopoietic differentiation, noting that multipotent progenitors, which derive from HSCs, generally lack or possess minimal self-renewal capabilities. This is in contrast to HSCs, which retain long-term self-renewal potential [5]. In a similar way, ref. [6] reported that Lin−/CD45−/Sca-1+, also known as very small embryonic-like stem cells (VSELs), show resistance to whole-body radiation due to their dormant state, unlike Lin−/CD45+/Sca-1+ hematopoietic cells, which are susceptible. Together, these studies stress the necessity of comprehending Lin− cell proliferation within the larger framework of hematopoietic progenitor activity and self-renewal capacity.

Surface markers like CD34, CD45, Sca-1, and c-Kit have been instrumental in isolating endothelial progenitor cells and HSC-enriched populations. These markers provide insights into the heterogeneity of HSCs and their potential in vascular and tissue regeneration [3]. Beyond intrinsic controls, external factors such as the HSC microenvironment and signaling pathways are equally vital to HSC proliferation. For instance, granulocyte colony-stimulating factor (G-CSF) has been shown to mobilize dormant HSCs without inducing proliferation, illustrating the complexity of regulatory mechanisms within the bone marrow niche [7]. Understanding Lin− cells’ function in HSC proliferation holds major relevance for advancing regenerative therapies, unraveling hematopoiesis, and crafting innovative treatments.

Past studies have indicated that certain compounds can stimulate HSC proliferation. Boron-based agents have been observed to enhance wound healing in vitro and in vivo, underlining their research value [8]. Hydrogels combining NaB and pluronic have demonstrated efficacy in promoting skin wound healing both in laboratory and live settings, positively influencing cellular movement and the healing process [8,9]. Furthermore, boric acid (BA) has been shown to assist in healing diabetic wounds and supporting tissue regeneration by boosting cell proliferation, migration, growth factor production, and inflammation modulation [10,11,12]. BA has also been found to upregulate gene and growth factor expression in dermal cells by encouraging angiogenesis [13,14]. These findings suggest that BA’s antimicrobial traits and effects on cellular functions contribute meaningfully to wound recovery.

Additionally, boric acid has been reported to reduce alveolar bone loss and promote osteoblast formation, highlighting its role in cell growth and bone development [15]. On a molecular level, it influences enzyme regulation, cell proliferation, growth, development, and energy metabolism [16]. Furthermore, boric acid has been associated with minimizing DNA double-strand breaks and expediting wound healing in human epithelial cells, indicating its role in DNA repair and cellular injury responses [17]. In conclusion, boric acid and its derivatives are deeply involved in various cellular processes. Their effects range from supporting wound repair and bone formation to modulating gene activity and enzyme function, positioning boric acid as a versatile and valuable component in stem cell biology.

Three boron compounds were selected for this study based on their distinct chemical properties and prior evidence of biological activity in mammalian cells. Boric acid (BA; H_3_BO_3_) is a simple, water-soluble boron-containing acid that exists as an undissociated molecule at physiological pH and has known wound-healing and anti-inflammatory properties. Sodium pentaborate pentahydrate (NaB; NaB_5_O_8_·5H_2_O) is a polymeric borate anion-containing salt that releases pentaborate ions upon dissolution. Sodium 2-pentaborate pentahydrate-8 (Na2B8; Na_2_B_8_O_13_·8H_2_O, also known as disodium octaborate tetrahydrate) contains octaborate anions and has distinct solubility and ionization characteristics. These structural differences may influence cellular uptake, intracellular speciation, and interaction with biomolecules such as enzymes, cell surface receptors, and signaling intermediates. We hypothesized that these chemical differences would translate into differential biological effects on HSPC populations, a hypothesis supported by our observed results showing that NaB preferentially expanded Sca-1+ cells while Na2B8 elevated LSK and LSKCD34^Low^ populations.

## 2. Materials and Methods

### 2.1. Ethical Approvals

All procedures involving animals were carried out in full compliance with ethical standards, under the oversight of the Animal Care and Use Ethical Committees at Yeditepe University. This study received ethical approval with the reference number 651.

### 2.2. Separation of Mouse Bone Marrow HSCs (Lin-Sca-1+c-Kit+CD34-/Low)

Hematopoietic stem cells from murine bone marrow (mBM-HSCs) were isolated from the femurs and tibias of Balb/c mice aged six to eight weeks, provided by YUDETAM, Turkey. The mice were euthanized humanely, and their bones sterilized using 70% ethanol. Bone marrow was extracted by flushing with ice-cold Dulbecco’s phosphate-buffered saline (DPBS), using a 26-gauge needle and syringe. After collection, the cells were centrifuged at 1500 rpm for five minutes and passed through a 70 μm cell strainer (BD Pharmingen, San Diego, CA, USA, Cat. No. 352350) [18,19,20].

### 2.3. Selection of Mouse Lin-Sca-1+c-Kit+ (mLSK) Cells

To enrich Lin− HSCs, a magnetic separation protocol established in earlier studies was followed [18,19,20]. The Mouse Hematopoietic Progenitor Cell Enrichment Kit (BD Pharmingen, San Diego, CA, USA, Cat. No. 558451) was used for this process. Cells were suspended in ice-cold DPBS supplemented with 2% fetal bovine serum (FBS, Sigma Aldrich, St. Louis, MO, USA, Cat. No. 12103C). Prior to antibody incubation, a mouse Fc block was applied to minimize non-specific binding. Cells were then incubated with a biotinylated antibody cocktail targeting lineage markers. After washing, streptavidin particles were added, and magnetic separation was conducted using the IMagnet system (BD Pharmingen, San Diego, CA, USA, Cat. No. 552311) to isolate Lin− cells. These purified cells were stained using PE-conjugated c-Kit (CD117) and PE-Cy7-conjugated Sca-1 antibodies to identify the LSK population. The labeled LSK cells were studied using an FACS ARIA cell sorter (BD Biosciences, San Diego, CA, USA, Cat. No. 23-11539-00).

### 2.4. Immunophenotypic Analysis of LSK Cells Post Boron Compound Exposure

The Lin− cell population was cultured in 96-well plates using StemSpan SFEM media, enriched with 1% PSA and a cocktail of human cytokines (StemSpan™ CC100, Stemcell Technologies, Vancouver, BC, Canada, Cat. No. 02690). All boron compounds utilized in the experiment were quantified and reported in µ molar (µM) concentration doses. These Lin− cells were treated with a range of concentrations (0.1 µM, 1 µM, 10 µM) of BA, NaB, and Na2B8 for 4 to 7 days. Post-treatment, the cells were labeled with mouse HSC-specific markers, including c-Kit+ (CD117) PE, CD34 FITC, Sca-1 (Stem cell antigen-1), PE-Cy7, and an APC-conjugated lineage marker cocktail as per BD Stem Flow (San Diego, CA, USA, Catalog No. 560492) specifications. Flow cytometric analysis was then conducted to ascertain the proportions of the LSK and LSKCD34^Low^ cell subsets as we have done before [18,19,20].

HSPCs were identified based on the LSK immunophenotype using flow cytometry. While this gating strategy is widely used for HSPC enrichment, it does not discriminate between long-term HSCs, short-term HSCs, and multipotent progenitors. Additional markers such as CD150 and CD48 (SLAM family) were not included in this study due to the constraints of the experimental design and antibody panel at the time of experimentation.

### 2.5. Evaluating the Impact of Boron Compounds on Lin− Cell Proliferation

Lin− cells were cultured in 96-well plates using StemSpan SFEM medium, enriched with 1% PSA and a proprietary blend of human cytokines (StemSpan™ CC100, Stemcell Technologies, Vancouver, BC, Canada, Cat. No. 02690). These Lin− cells were treated for 4 days with varying concentrations (0.1 µM, 1 µM, and 10 µM) of BA and NaB. Subsequently, the cell counts of the Lin− population were determined using the Cytell™ Cell Imaging System (GE Healthcare Life Sciences, Marlborough, MA, USA, Cat. No: 29-0567-49).

All experiments were conducted with three independent biological replicates, each derived from different individual mice. For each biological replicate, three technical replicates per treatment condition were performed to ensure consistency and reproducibility of the results. The relatively high standard deviations observed in some treatment groups are attributable to biological heterogeneity inherent in primary hematopoietic stem cell populations and their differential response to boron compound treatment.

### 2.6. Statistical Analysis

All data are presented as mean ± standard deviation (SD). For comparisons between two groups, statistical significance was assessed using a two-sided unpaired Student’s *t*-test, with *p*-values *p* < 0.05 considered statistically significant (* *p* < 0.05, ** *p* < 0.01, *** *p* < 0.001). For this study, the *t*-test was selected over one-way ANOVA due to the specific aim of comparing each treatment group directly with a single control group (PBS). Since the experimental design did not require comparison among all treatment groups simultaneously, but rather individual comparisons to control, the two-sample *t*-test was deemed appropriate and statistically valid.

## 3. Results

### 3.1. Impact of Boron Compounds on Mouse HSC Immunophenotyping

**Figure 1:**
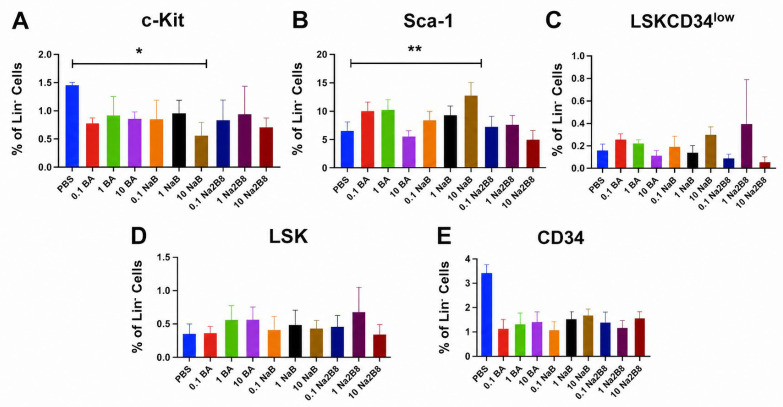
The bar graph series represents the percentage of Lin− cells expressing specific markers after treatment with various concentrations of BA, NaB, and Na; (**A**) C-Kit+ expression, (**B**) Sca-1+ marker expression, (**C**) LSKCD34^Low^ expression, (**D**) LSK expression, and (**E**) CD34+ expression shown in Lin− cells. Error bars indicate the standard deviation of the mean (* *p* < 0.05, ** *p* < 0.01). (LSK: Lin-Sca-1+c-Kit+. This population includes both HSCs and multipotent progenitors) (*n* = 3).

As shown in Figure 1A–E, the expression of c-Kit+ (CD117), Sca-1, and CD34+ markers in hematopoietic cells were examined after treating Lin− cells isolated from mice with various boron derivatives. C-Kit+ (CD117) and CD34+ markers either present stem or progenitor cells and play a critical role in the growth and differentiation of these cells. In the PBS-treated control group, the percentage of c-Kit+ (CD117) was higher, whereas this percentage decreased in the groups treated with boron derivatives. This suggests that boron derivatives may exert an unexpected suppressive effect on the c-Kit+ (CD117) progenitor populations. In Figure 1B, the expression of Sca-1+, a hematopoietic stem cell marker, showed a statistically significant increase at a 10 µM NaB dose compared to the PBS group (** *p* < 0.01). This finding suggests that higher doses of NaB may enhance the expression of Sca-1+ in HSCs. Although no statistically significant increase was observed in other groups, some demonstrated biologically notable increases. In Figure 1C, the LSKCD34^Low^ cells, representing a more immature fraction of the hematopoietic stem cell population, were observed. In this graph, the boron derivatives displayed varying effects on these cells. The 0.1 µM BA group increased compared to PBS. Similarly, the 0.1 µM and 10 µM NaB doses also increased the expression, but these increases were not statistically significant. However, slight increases were noted in the 10 µM NaB and 1 µM Na2B8 groups. The LSK cells, shown in Figure 1D, represent the hematopoietic stem and progenitor cell population. No statistically significant difference was observed between the groups regarding LSK cell percentages. This may suggest that the LSK population is inherently more stable and less responsive to boron derivatives at the tested concentrations. In Figure 1E, the responses of the CD34+ cell population, which represents a rather differentiated hematopoietic progenitor, to boron derivatives were examined. There was a reduction in this population but with no statistically significant difference compared to PBS treatment. This suggests that the effects of boron derivatives on this population may be limited and biologically subtle.

### 3.2. Effect of Boron Compounds on Lin− Cell Growth

**Figure 2:**
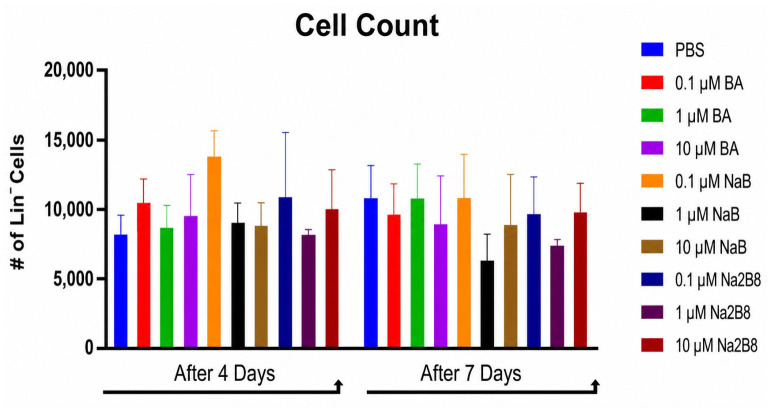
The bar graph represents the cell count of Lin− cells four and seven days post-treatment with various concentrations of BA, NaB, and Na2B8. The cell counts are compared to PBS control. Error bars indicate the standard deviation from the mean (*n* = 3).

The results of hematopoietic cell counts on days 4 and 7 after treatment with boron derivatives are shown in Figure 2. On day 4 post-treatment, a noticeable increase is observed in the 0.1 µM NaB group compared to the control group; however, this difference is not statistically significant. This biological increase may suggest that boron derivatives at certain doses could promote cell proliferation. Similarly, a visible increase is noted in the 0.1 µM BA group compared to the PBS group, but given that this increase is not statistically significant, it can be suggested that BA at low doses may have the potential to biologically enhance cell numbers. Likewise, in the 0.1 µM Na2B8 group, a visible increase is observed compared to the PBS group, although this increase is not statistically significant. On day 7 post-treatment, an increase in cell numbers was observed in the PBS group. However, no increase in cell numbers was observed in any of the boron derivative-treated groups compared to the PBS group, and in some groups, a reduction in cell numbers was noted. The discrepancy between days 4 and 7 may be due to boron derivatives supporting short-term cell proliferation while their long-term effects are limited or even detrimental. Additionally, the cells’ adaptive response to boron derivatives might have altered over time. Boron derivatives have shown certain biological effects on hematopoietic cell proliferation compared to PBS, but additional experiments and higher sample sizes may be required for a clearer understanding of this effect.

### 3.3. Impact of Boron Compound Combinations on Mouse HSC Immunophenotyping

**Figure 3:**
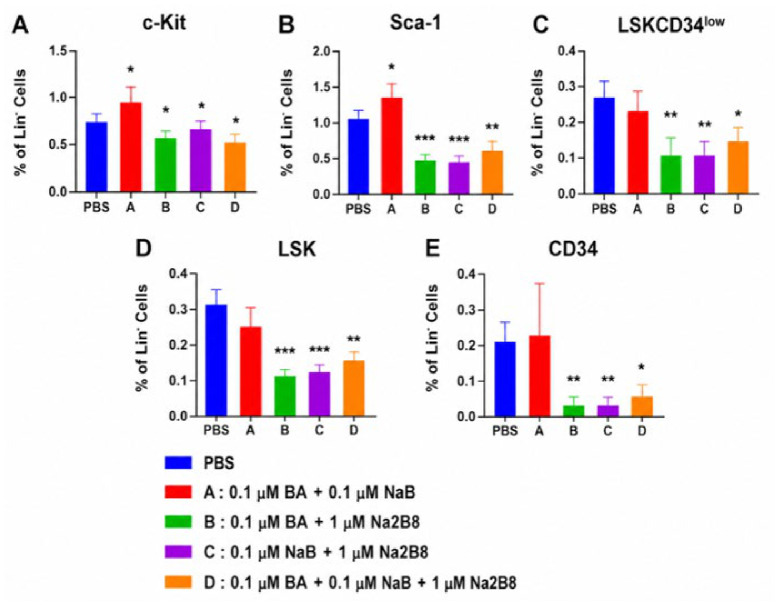
The bar graph series represents the percentage of Lin− cells expressing specific markers after treatment with various combinations of BA, NaB, and Na2B8; (**A**) c-Kit+ expression, (**B**) Sca-1+ marker expression, (**C**) LSKCD34^Low^ expression, (**D**) LSK expression, and (**E**) CD34+ expression. The standard deviation of the mean is shown with error bars (* *p* < 0.05, ** *p* < 0.01, *** *p* < 0.001) (*n* = 3).

The effective concentrations of boron derivatives on Lin− cells were tested and presented in combination forms in Figure 3. In Figure 3A,B, the combination of 0.1 µM BA + 0.1 µM NaB shows a statistically significant proliferative effect on c-Kit+ (CD117) and Sca-1+ cells (* *p* < 0.05), while the other combinations are observed to decrease the cell ratios (* *p* < 0.05, ** *p* < 0.01, *** *p* < 0.001). In Figure 3C,D, a statistically significant reduction in cell ratios of LSK and LSKCD34^Low^ cells, rather than proliferation, is observed across other combinations (* *p* < 0.05, ** *p* < 0.01, *** *p* < 0.001). Notably, CD34+ differentiated progenitors were significantly lowered post treatment of B, C, and D combinations except A, which included 0.1 µM BA and 0.1 µNaB (Figure 3E).

**Figure 4:**
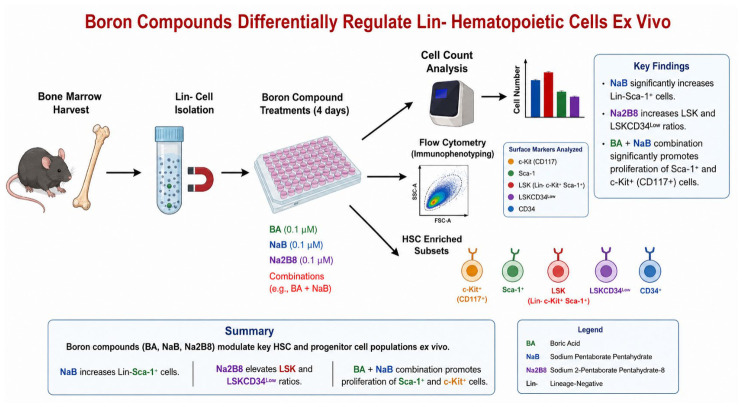
Boron compounds differentially regulate ex vivo expansion and immunophenotypic modulation of Lin− hematopoietic stem/progenitor cells. Mouse bone marrow-derived Lin− cells were magnetically isolated and treated ex vivo with boric acid (BA), sodium pentaborate pentahydrate (NaB), sodium 2-pentaborate pentahydrate-8 (Na2B8), or their combinations for 4 days. Following treatment, cell proliferation and hematopoietic stem cell-associated marker expression were evaluated by cell counting and flow cytometric immunophenotyping. Boron compounds exerted distinct effects on stem and progenitor cell populations, including c-Kit+ (CD117), Sca-1+, LSK, LSKCD34^Low^, and CD34+ subsets. NaB treatment significantly increased the Lin-Sca-1+ population, whereas Na2B8 elevated LSK and LSKCD34^Low^ fractions. In addition, the combined treatment of 0.1 µM BA + 0.1 µM NaB significantly promoted proliferation and expansion of Sca-1+ and c-Kit+ (CD117+) cells. These findings suggest that boron derivatives enhance ex vivo HSC expansion through modulation of stem cell-associated populations and potentially through metabolic pathways linked to HSC self-renewal.

The biological effects of different boron derivatives on HSCs vary depending on the combinations. In particular, 0.1 µM BA + 0.1 µM NaB and similar combinations show proliferative effects on HSCs with certain cell surface markers, while other combinations can suppress cell growth (Figure 4). This may be due to the differences in cellular mechanisms in the specific responses of HSCs to boron derivatives and concentrations.

## 4. Discussion

HSCs play a critical role in treating hematological disorders and malignancies through HSC transplantation due to their self-renewal capacity and ability to differentiate into different cell types [21]. Although umbilical cord blood and mobilized peripheral blood (PB) have been investigated as alternative HSC sources to bone marrow, the limited amount of HSC they contain may limit the success of transplantation and related therapeutic processes. To overcome this challenge, small molecules have emerged as an effective approach to increasing HSC numbers [22,23,24,25]. To fill this gap, we applied boron derivatives in vitro to increase the number of HSCs in this study. In this study, we aimed to investigate the potential effects of boron derivatives on mouse stem cells in vitro as a strategy to increase the number of HSCs. With this approach, we aimed to fill the existing gaps in the expansion of HSCs.

This study demonstrates that the effects of different boron derivatives on HSC-enriched populations vary depending on concentration and combination. Specifically, the combination of 0.1 µM BA + 0.1 µM NaB showed a significant proliferative effect on c-Kit+ (CD117) and Sca-1+ cells, while other combinations generally reduced cell numbers. While the combination of 0.1 µM BA + 0.1 µM NaB emerged as a potential candidate for promoting c-Kit+ or Sca-1+ hematopoietic progenitor expansion, the most effective boron derivative overall was 10 µM NaB for Sca-1+ and to some extend LSKC34^Low^ cells. Moreover, certain Na2B8 treatments enhanced proliferation in LSKCD34^Low^ cells. These findings highlight the proliferative effects of boron derivatives at various doses and in combination, suggesting that further research is needed to explore their mechanisms and long-term effects on HSCs.

The diverse functions of boron compounds in stem cell biology and tissue regeneration have attracted growing interest due to their ability to affect cell growth and differentiation. In recent years, a range of approaches has been explored to expand hematopoietic stem cells in culture, including specialized culture systems, supplementation with cytokines and growth factors, and the incorporation of small molecules. One such molecule, 2-aminoethoxydiphenyl borate (2-APB) inhibitor of inositol 1,4,5-trisphosphate receptors (InsP3R) has shown promise in promoting the proliferation of human HSCs. Notably, 2-APB was more effective in expanding human umbilical cord-derived HSCs in vitro than mobilized peripheral blood HSCs [20,26,27,28]. Our findings with BA, NaB, and Na2B8 may similarly involve calcium-dependent mechanisms, though this requires direct investigation. We hypothesize that NaB may activate calcium-dependent signaling pathways given its structural similarity to known calcium modulators, while Na2B8 may influence distinct transcriptional programs regulating LSK maintenance. The synergistic effect of the BA+NaB combination suggests potential complementary mechanisms, possibly involving both extracellular calcium sensing and intracellular borate-mediated enzyme modulation.

In line with Demirci and colleagues, our findings showed that treatment with NaB led to a marked increase in Lin− cell proliferation [13]. That study had demonstrated NaB’s ability to boost cell proliferation and migration, stimulate growth factor expression, and reduce inflammation—an effect beneficial for healing diabetic wounds. These findings suggest that boron compounds may exert biological effects that extend well beyond HSC regulation, offering promising applications in wound healing and tissue regeneration.

Moreover, the targeted effects we observed on c-Kit+ and Sca-1+ cell subpopulations reflect the intricate regulation of HSCs described in prior studies. For example, ref. [7] discussed the fine-tuned control mechanisms within the HSC niche, illustrating how granulocyte colony-stimulating factor (G-CSF) mobilizes dormant HSCs without triggering their proliferation. Our research builds on this understanding by showing that specific boron compounds can differentially affect subtypes of stem cells—echoing insights from [29], who identified transcription factor Zfp90 as a key regulator of HSC proliferation.

Additionally, the combined use of sodium borate and boric acid may have yielded synergistic or additive effects, pointing to the potential for designing new strategies for HSC expansion. This is particularly significant for fields like regenerative medicine and bone marrow transplantation, where reliable and efficient HSC expansion remains a major obstacle. Our findings contribute to the broader effort in stem cell science to identify compounds that can favorably modify the stem cell niche and associated signaling pathways to enhance ex vivo expansion. This aligns with the various documented roles of boron compounds in encouraging both osteogenic and odontogenic differentiation and in promoting wound repair [9,14].

The biological impacts of boric acid and its derivatives go beyond merely promoting cell proliferation, influencing numerous aspects of human physiology. Boric acid is mainly absorbed through the gastrointestinal and respiratory tracts. Research has demonstrated that boron—the active component in boric acid—accumulates at low concentrations in different tissues, suggesting effective absorption and systemic distribution within the body [30]. Notably, boric acid may also act as an antioxidant by limiting the formation of reactive oxygen species, thereby offering cellular protection against oxidative stress [31]. This antioxidant property could be particularly valuable in metabolic conditions marked by increased oxidative damage.

Beyond these effects, boric acid has exhibited antiproliferative properties in certain cancer cell lines. For example, it has been shown to trigger apoptosis in glioblastoma cells through pathways involving endoplasmic reticulum stress and inflammatory responses [32]. In addition to its anticancer activity, boron compounds have demonstrated the ability to regulate cell signaling and metabolic pathways that are relevant in managing conditions like obesity and diabetes [33]. Nutritionally, while boron is not officially recognized as an essential trace element, observational studies indicate that boric acid and its derivatives may affect nutrient metabolism and enhance the bioavailability of key vitamins and minerals [34].

Another investigation explored the effects of boron derivatives—sodium pentaborate pentahydrate (SPP) and sodium perborate tetrahydrate (SPT)—on breast cancer cell lines. The findings revealed that both compounds inhibited cell proliferation and induced apoptosis. They also increased PD-L1 protein levels and influenced T-cell activity, suggesting that boron derivatives may contribute not only to suppressing tumor growth but also to modulating immune responses in cancer therapy [35]. In addition, previous findings have indicated that boric acid has cytotoxic and pro-apoptotic effects on leukemic cells [36]. We have recently shown that MEIS1 inhibitors can reduce the activity of leukemic stem cells while potentially increasing their susceptibility to chemotherapy [37]. Thus, future studies could benefit from the evaluation of the combined use of these compounds alongside standard therapies to better define their clinical value.

In a separate study on hematopoietic stem cells, researchers evaluated both normal and Trp53-deficient mice, using SLAM (Signaling Lymphocyte Activation Molecule) and LSK markers for HSC isolation. The study found that SLAM markers were effective for enriching HSC populations, and that Trp53 deficiency enhanced self-renewal capacity. However, this increased self-renewal did not translate into improved engraftment capability [38]. Other studies aiming to expand HSC numbers have tested small molecules such as TEPA, SR1, nicotinamide, and UM171, with some progressing to clinical trials. These studies also emphasized the importance of key signaling pathways like Wnt and Notch in determining HSC fate and self-renewal potential [39,40].

Despite the promising results regarding the stimulatory effects of boron compounds on hematopoietic stem and progenitor cell populations, this study does have limitations. Firstly, the functional capacity of the expanded progenitor cells was not assessed through colony-forming unit (CFU) assays, which are essential for verifying hematopoietic potential in vitro. Secondly, the long-term engraftment and differentiation capacity of the expanded HSCs were not evaluated using in vivo transplantation models with extended follow-up periods. Additionally, our immunophenotypic analysis relied on conventional LSK gating without SLAM markers, which limited precise discrimination of LT-HSCs from progenitors. It is critical to note that the LSK compartment includes multipotent progenitors that lack long-term self-renewal capacity. Thus, our findings demonstrate modulation of HSC-enriched populations rather than definitive expansion of functional LT-HSCs. Furthermore, while we hypothesize that boron compounds may influence cellular metabolism based on the prior literature, our study did not include direct metabolic measurements (e.g., mitochondrial function, glycolysis, ROS levels, or metabolomics). Therefore, claims regarding metabolic modulation remain speculative and require dedicated investigation. Finally, tested concentrations of boron derivatives may not directly reflect clinically achievable conditions, and biological variability among replicates further emphasizes the need for larger sample sizes.

## 5. Conclusions

This study demonstrates that boron compounds BA, NaB, and Na2B8 differentially modulate immunophenotypic markers of mouse HSC-enriched populations ex vivo. NaB increased Lin-Sca-1+ cells, Na2B8 elevated LSK and LSKCD34^Low^ ratios, and the combination BA+NaB promoted proliferation of Sca-1+ and c-Kit+ cells. However, these findings are based on phenotypic analysis without functional validation; therefore, they should be interpreted as changes in HSC-enriched fractions rather than definitive expansion of functional long-term HSCs. Further studies incorporating CFU assays, transplantation models, and SLAM marker profiling are warranted to assess the true stem cell potential of boron-compound-expanded populations and to elucidate the underlying mechanisms.

In conclusion, this study not only reinforces the role of boron compounds in modulating cell growth and proliferation but also opens new research directions to uncover the mechanisms through which they influence hematopoietic stem cell biology. Future studies should aim to elucidate these mechanisms in detail, potentially unlocking new therapeutic strategies for hematological disorders, tissue injuries, and degenerative diseases. In summary, this study advances our understanding of HSC biology and highlights boron compounds as promising candidates for ex vivo stem cell expansion strategies in regenerative medicine.

## Data Availability

The original contributions presented in this study are included in the article. Further inquiries can be directed to the corresponding author.
